# Complete genome sequence of *Vibrio campbellii* strain 20130629003S01 isolated from shrimp with acute hepatopancreatic necrosis disease

**DOI:** 10.1186/s13099-017-0180-2

**Published:** 2017-06-01

**Authors:** Xuan Dong, Hailiang Wang, Peizhuo Zou, Jiayuan Chen, Zhen Liu, Xuepeng Wang, Jie Huang

**Affiliations:** 1 Yellow Sea Fisheries Research Institute, Chinese Academy of Fishery Sciences, Laboratory for Marine Fisheries Science and Food Production Processes, Qingdao National Laboratory for Marine Science and Technology, Key Laboratory of Maricultural Organism Disease Control, Ministry of Agriculture, Qingdao Key Laboratory of Mariculture Epidemiology and Biosecurity, Qingdao, China; 2 Shanghai Ocean University, Shanghai, China; 3 Shandong Agricultural University, Taian, China; 4 Shanghai Majorbio Bio-pharm Biotechnology, Shanghai, China; 5 Institute of Oceanology, Chinese Academy of Sciences, Qingdao, China

## Abstract

**Background:**

*Vibrio campbellii* is widely distributed in the marine environment and is an important pathogen of aquatic organisms such as shrimp, fish, and mollusks. An isolate of *V. campbellii* carrying the *pirAB* ^*vp*^ gene, causing acute hepatopancreatic necrosis disease (AHPND), has been reported. There are no previous reports about the complete genome of *V. campbellii* causing AHPND (VC_AHPND_). To extend our understanding of the pathogenesis of VC_AHPND_ at the genomic level, the genome of *V. campbellii* 20130629003S01 isolated from a shrimp with AHPND was sequenced and analysed.

**Results:**

The complete genome sequence of *V. campbellii* 20130629003S01 was generated using the PacBio RSII platform with single molecule, real-time sequencing. The 20130629003S01 strain consists of two circular chromosomes (3,621,712 bp in chromosome 1 and 2,245,751 bp in chromosome 2) and four plasmids of 70,066, 204,531, 143,140, and 86,121 bp. The genome contains a total of 5855 protein coding genes, 134 tRNA genes and 37 rRNA genes. The average nucleotide identity value of 20130629003S01 and other reference *V. campbellii* strains was 97.46%, suggesting that they are closely related.

**Conclusions:**

The genome sequence of *V. campbellii* 20130629003S01 and its comparative analysis with other *V. campbellii* strains that we present here are important for a better understanding of the genomic characteristics of VC_AHPND_.

**Electronic supplementary material:**

The online version of this article (doi:10.1186/s13099-017-0180-2) contains supplementary material, which is available to authorized users.

## Background

*Vibrio campbellii* is widely distributed in the marine environment and is an important pathogen of wild and reared marine organisms such as shrimp, fish, and mollusks [1]. In recent years, an isolate of *V. campbellii* carrying the *pirAB* ^*vp*^ gene that causes acute hepatopancreatic necrosis disease (AHPND) has been reported [2]. Shrimp production in AHPND-affected regions (SE Asia and Mexico) has dropped sharply, which is causing heavy economic losses. Initially, *V. parahaemolyticus*, which becomes virulent by acquiring a unique extrachromosomal AHPND-associated plasmid carrying *pirAB* ^*vp*^ (VP_AHPND_), was the only pathogen known to cause AHPND. Later, non-*V. parahaemolyticus* AHPND-causing *Vibrio* started emerging, and *V. harveyi*-like, *V. owensii* and *V. campbellii* strains have been reported [2–4].

Recent studies have shown that VP_AHPND_ possesses not only toxin genes but also a ~70 kb plasmid, which expresses Pir^*vp*^ [5]. However, there are no reports about the complete genome of *V. campbellii* that causes AHPND (VC_AHPND_). In this paper, we obtained the complete genome sequence of one strain of *V. campbellii*, which was isolated in June 2013 from the hepatopancreas of diseased *Litopenaeus vannamei* in Guangxi, China. PCR amplifications were performed using VpPirA and VpPirB primers specific to the *pirAB* ^*vp*^ genes (*pirA* ^*vp*^ and *pirB* ^*vp*^), and this strain was evaluated for its pathogenicity in *L. vannamei* [2]. The shrimp showed typical symptoms of AHPND, and cumulative mortalities reached 100% [2]. The genome sequencing of 20130629003S01 provides timely information for a better understanding of the genomic characteristics of the pathogen.

## Methods

### Genomic DNA isolation, sequencing and assembly

Strain 20130629003S01 is *V. campbellii* isolated in June 2013 from AHPND-affected *L. vannamei* in Guangxi, China. The genomic DNA of this strain was extracted using the Wizard Genomic DNA Purification Kit (Promega, Madison, WI, USA). The DNA was examined by 1% agarose gel electrophoresis and quantified using a NanoDrop 2000 spectrophotometer (Thermo Scientific, MA, USA). The genomic DNA was sequenced using the PacBio RSII platform by Majorbio Bio-Pharm Biotechnology Co., Ltd., Shanghai, China. A 10-kb DNA library was constructed according to the manufacturer’s protocols and sequenced using single-molecule real-time (SMRT) sequencing technology with P6-C4 chemistry. One SMART cell was used for sequencing, and the data were assembled de novo using the hierarchical genome assembly process (HGAP) [6]. The assembly was based on 1.02 Gb of PacBio data and polished with three successive passes through Quiver to reach a final consensus accuracy at 194× coverage. This assembly consisted of six contigs including two chromosomes and four plasmids. The repeat sequences at the end of the six contigs were removed to obtain the complete genome and plasmid sequences.

### Genome annotation

Gene prediction was carried out using Glimmer [7], while rRNA and tRNA were analysed using RNAmmer [8] and tRNAscan-SE version 1.21 [9]. Gene annotation was carried out based on homology searches against the gene ontology (GO) database and clusters of orthologous groups (COG) protein database. Prophage regions were identified using the PHAge Search Tool (PHAST) [10]. Virulence genes were searched for using the virulence factor of pathogenic bacteria database (VFDB) [11] and BLAST.

### Comparative genome analysis

The complete reference genome sequences of *V. campbellii* strains were downloaded from NCBI and used for comparative genome analysis. The accession numbers of the reference *V. campbellii* strains were DS40M4 (AGIE01000001–AGIE01000121), CAIM_519 (AMDG01000001–AMDG01000213), BAA-1116 (CP006605–CP006607), UMTGB204 (JSFE01000001–JSFE01000060), HY01 (DS179406–DS179608), LMB29 (CP019293–CP019298) and 051011E (BBKU01000001–BBKU01000219). The JSpecies program was used for calculating the average nucleotide identity (ANI) value [12] among the 8 strains, which were cut into fragments of 1020 bp for calculating the ANI values by using the BLAST algorithm [13]. Next, a distance dendrogram was constructed using the R program.

### Quality assurance

The genomic DNA used for sequencing was isolated from a pure culture of *V. campbellii* strain 20130629003S01. The 16S rRNA gene was amplified and sequenced, and BLAST was performed against the NCBI database.

## Initial findings

### Genome properties

**Fig. 1:**
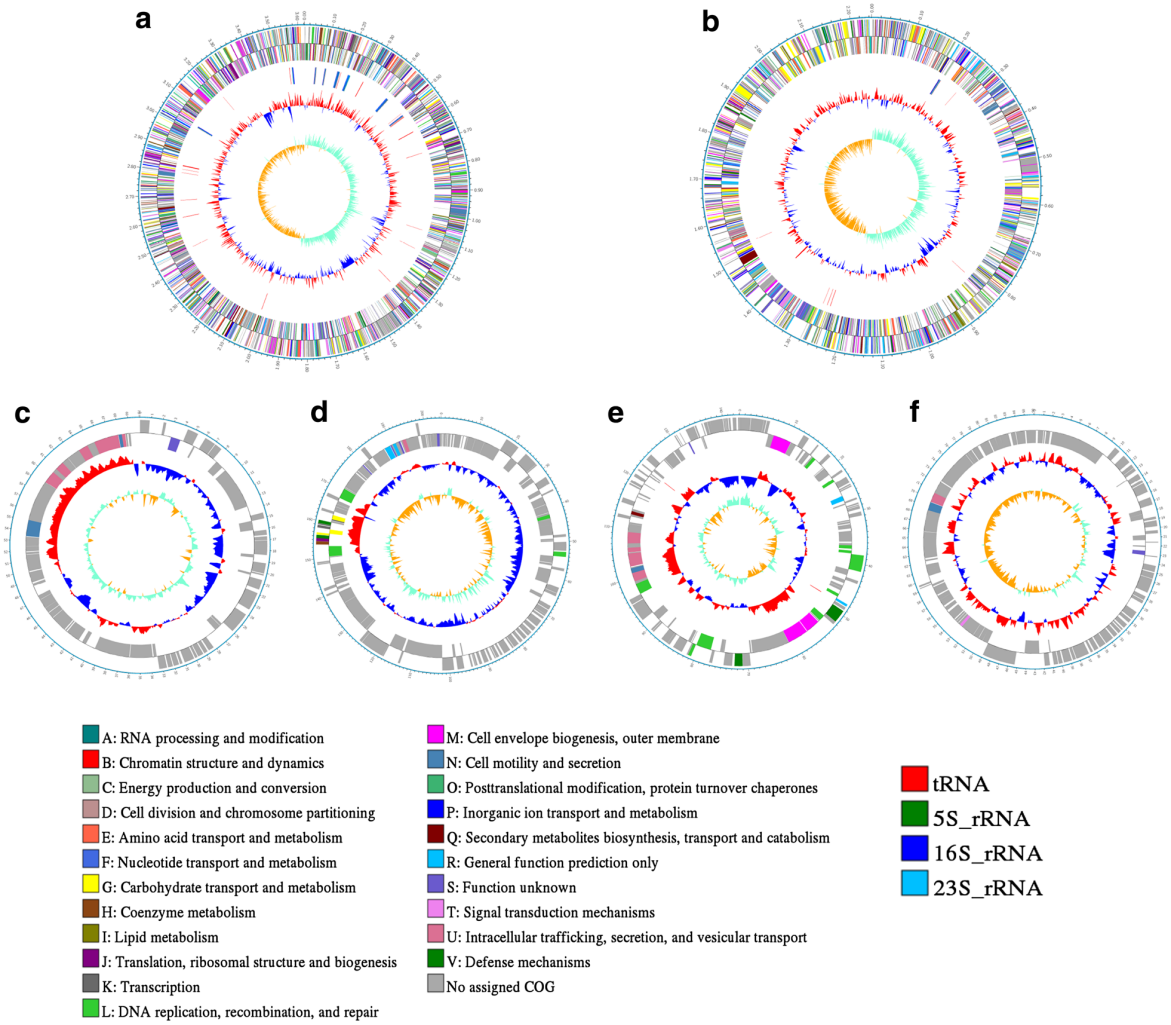
The circular genome maps of the strain 20130629003S01. **a** Chromosome 1. **b** Chromosome 2. **c** Plasmid 1. **d** Plasmid 2. **e** Plasmid 3. **f** Plasmid 4. The tracks from outside to inside represent the identity of the genome size, CDS (+), CDS (−), rRNA and tRNAs, GC contents, GC skews. Between these circles, all annotated ORFs were colored differently according to the COG assignments

The complete genome of *V. campbellii* strain 20130629003S01 includes two circular DNA chromosomes of 3,621,712 and 2,245,751 bp with GC content of 45.26–45.56%, and four plasmids of 70,066, 204,531, 143,140, and 86,121 bp with GC content of 39.56–45.90%. chromosome 1 consists of 3216 protein coding genes, 34 rRNA genes and 117 tRNA genes. Chromosome 2 consists of 2057 protein coding genes, 3 rRNA genes and 15 tRNA genes. Plasmid 1 consists of 86 protein coding genes. Plasmid 2 consists of 229 protein coding genes. Plasmid 3 consists of 145 protein coding genes and 2 tRNA genes. Plasmid 4 consists of 122 protein coding genes. Information about the features of the complete genomic sequence of *V. campbellii* strain 20130629003S01 is provided in Fig. 1. The genome of this strain contains four incomplete phage sequences on chromosome 1, one incomplete phage sequence on chromosome 2, and one intact phage sequence on chromosome 2.

### Functional categorization

The results of COG categorization of the predicted open reading frames (ORFs) are shown in Additional file 1: Figure S1. The ORFs could be categorized into 22 classes, which include S (424 ORFs, function unknown), E (339, amino acid transport and metabolism), K (293 ORFs, transcription), R (273 ORFs, general function prediction only), T (262 ORFs, signal transduction mechanisms), G (256 ORFs, carbohydrate transport and metabolism), P (253 ORFs, inorganic ion transport and metabolism), C (236 ORFs, energy production and conversion), M (229 ORFs, cell wall/membrane/envelope biogenesis), J (190 ORFs, translation, ribosomal structure and biogenesis), O (171 ORFs, post-translational modification, protein turnover, chaperones), L (163 ORFs, replication, recombination and repair), H (131 ORFs, coenzyme transport and metabolism), U (128 ORFs, intracellular trafficking, secretion, and vesicular transport), N (122 ORFs, cell motility), I (101 ORFs, lipid transport and metabolism), F (90 ORFs, nucleotide transport and metabolism), Q (73 ORFs, secondary metabolites biosynthesis, transport and catabolism), V (68 ORFs, defence mechanisms), D (37 ORFs, cell cycle control, cell division, chromosome partitioning), A (1 ORF, RNA processing and modification), and B (1 ORF, chromatin structure and dynamics).

### Pathogenesis and virulence factors

Key virulence factors in AHPND-causing *V. campbellii* are *pirAB* ^*vp*^ (*pirA* ^*vp*^ and *pirB* ^*vp*^) [2, 5]. The strain 20130629003S01 harbours four plasmids, pVCGX1, pVCGX2, pVCGX3, and pVCGX4. Interestingly, pVCGX1 has a *pirA* ^*vp*^ gene and *pirB* ^*vp*^ gene related to AHPND, and both *pirA* ^*vp*^ and *pirB* ^*vp*^ of pVCGX1 share 100% sequence identities with their orthologues in the plasmids pVA1 and pVPA3-1 of *Vibrio parahaemolyticus* [5, 14]. Therefore, pVCGX1 may contribute to pathogenesis.

### Comparative genome analysis

**Fig. 2:**
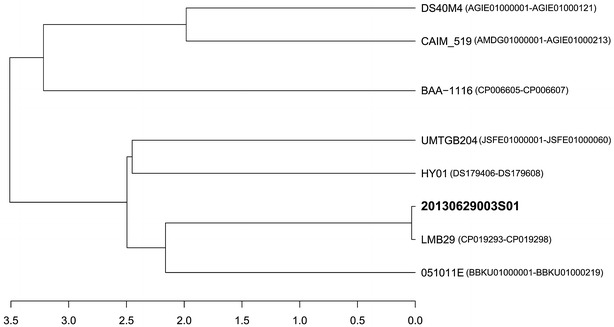
Distance dendrogram among *Vibrio campbellii* strains based on ANI values. The complete genome sequences of *V. campbellii* (DS40M4, CAIM_519, BAA_1116, UMTGB204, HY01, 20130629003S01, LMB29 and 051011E) revealed that the LMB29 strain has the closest evolutionary relationship (ANI value 98.53) with an isolate LMB29 from cage-cultured red drum with skin ulcer in China

Seven complete reference genome sequences of *V. campbellii* and their annotations were collected from the GenBank database. The ANI values were calculated using 8 strains, and all values between every two strains were greater than 95%. Furthermore, the 20130629003S01 strain was found to cluster with the LMB29 strain (Fig. 2). The LMB29 strain (GenBank accession number: CP019293.1) was isolated from cage-cultured red drum with skin ulcers in China. Comparative data are shown as a dendrogram in Fig. 2 and tabulated in Additional file 1: Table S1.

### Future directions

In conclusion, we report the 5.3 Mbp complete genome sequence of *V. campbellii* strain 20130629003S01. Additional comparative studies of the genomes of AHPND-causing *Vibrio* with the genome sequence of strain 20130629003S01 should provide genomic insights into the pathogenicity and virulence mechanisms of VC_AHPND_.

## Additional file

**Additional file 1: Figure S1.** Functional categorization of 20130629003S01 based on the COG database. **Table S1.** ANI values among *Vibrio campbellii* strains.

## Abbreviations

*L. vannamei*: *Litopenaeus vannamei*
ANI: average nucleotide identity
rRNA: ribosomal RNA
tRNA: transport RNA
COG: cluster of orthologous groups
GO: gene ontology
BLAST: basic local alignment search tool
PHAST: PHAge Search Tool
VFDB: virulence factor of pathogenic bacteria database
ORFs: open reading frames
