# Supplementary material for: Complete genome sequence of Vibrio campbellii strain 20130629003S01 isolated from shrimp with acute hepatopancreatic necrosis disease
Source: Gut Pathog. 2017 Jun 1;9:31. doi: 10.1186/s13099-017-0180-2 (PMC5452529; doi:10.1186/s13099-017-0180-2)
Supplement: Supplementary file 1 — Additional file 1: Figure S1. Functional categorization of 20130629003S01 based on the COG database. Table S1. ANI values among Vibrio campbellii strains. [file 13099_2017_180_MOESM1_ESM.docx]

**Additional Files**

**Complete genome sequence of *Vibrio campbellii* 20130629003S01 strain isolated from shrimp with acute hepatopancreatic necrosis disease**

Xuan Dong^1^, Hailiang Wang^1,5^, Peizhuo Zou^1,2^, Jiayuan Chen^1,3^, Zhen Liu^4^, Xuepeng Wang^3^, Jie Huang^1^*

^1^ Qingdao Key Laboratory of Mariculture Epidemiology and Biosecurity; Key Laboratory of Maricultural Organism Disease Control, Ministry of Agriculture; Function Laboratory for Marine Fisheries Science and Food Production Processes, Qingdao National Laboratory for Marine Science and Technology; Yellow Sea Fisheries Research Institute, Chinese Academy of Fishery Sciences, Qingdao, China;

^2^ Shanghai Ocean University, Shanghai, China

^3^ Shandong Agricultural University, Taian, China

^4^ Shanghai Majorbio Bio-pharm Biotechnology, Shanghai, China

^5^ Institute of Oceanology, Chinese Academy of Sciences, Qingdao, China

***Corresponding author.** Tel.: +86-0532-85823062; Fax: +86-0532-85811514; E-mail: huangjie@ysfri.ac.cn (Jie Huang).

**Figure S1**


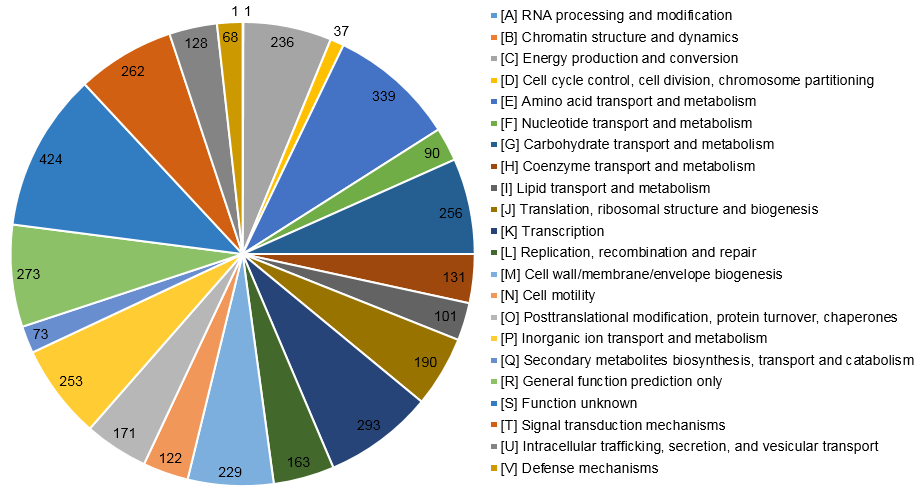


**Figure S1** Functional categorization of 20130629003S01 based on the COG database.

**Table S1. ANI values among *Vibrio campbellii* strains**

|  | 051011E | LMB29 | **20130629003S01** | HY01 | UMTGB204 | BAA-1116 | CAIM_519 | DS40M4 |
| --- | --- | --- | --- | --- | --- | --- | --- | --- |
| 051011E | 100 | 97.80 | 97.87 | 97.48 | 97.54 | 96.81 | 96.30 | 96.37 |
| LMB29 | 97.80 | 100 | 99.97 | 97.53 | 97.46 | 96.64 | 96.23 | 96.24 |
| **20130629003S01** | 97.87 | 99.97 | 100 | 97.56 | 97.46 | 96.73 | 96.30 | 96.29 |
| HY01 | 97.48 | 97.53 | 97.56 | 100 | 97.55 | 96.60 | 96.18 | 96.17 |
| UMTGB204 | 97.54 | 97.46 | 97.46 | 97.55 | 100 | 96.77 | 96.30 | 96.33 |
| BAA-1116 | 96.81 | 96.64 | 96.73 | 96.60 | 96.77 | 100 | 96.81 | 96.78 |
| CAIM_519 | 96.30 | 96.23 | 96.30 | 96.18 | 96.30 | 96.81 | 100 | 98.02 |
| DS40M4 | 96.37 | 96.24 | 96.29 | 96.17 | 96.33 | 96.78 | 98.02 | 100 |
